# Late morning biting behaviour of *Anopheles funestus* is a risk factor for transmission in schools in Siaya, western Kenya

**DOI:** 10.1186/s12936-023-04806-w

**Published:** 2023-11-30

**Authors:** Seline Omondi, Jackline Kosgei, George Musula, Margaret Muchoki, Bernard Abong’o, Silas Agumba, Caroline Ogwang, Daniel P. McDermott, Martin J. Donnelly, Sarah G. Staedke, Jonathan Schultz, Julie R. Gutman, John E. Gimnig, Eric Ochomo

**Affiliations:** 1 Entomology Section, Centre for Global Health Research, Kenya Medical Research Institute, P.O. Box 1578-40100, Kisumu, Kenya; 2 Department of Vector Biology, Liverpool School of Tropical Medicine, Pembroke Place, Liverpool, L3 5QA UK; 3 Division of Parasitic Diseases and Malaria, Centers for Disease Control and Prevention, Atlanta, GA 30333 USA

**Keywords:** *Anopheles funestus*, Malaria, Behaviour, Primary schools

## Abstract

**Background:**

Children in Kenya spend a substantial amount of time at school, including at dawn and dusk when mosquitoes are active. With changing vector behaviour towards early morning biting, it is important to determine whether there is an additional risk of transmission in schools. This study sought to understand whether late morning biting by *Anopheles funestus*, previously documented in households in western Kenya, was replicated in schools.

**Methods:**

From the 4th to the 6th of August 2023, human landing collections were conducted hourly in four schools in Alego Usonga sub-County, Siaya County. The collections were conducted in and outside five classrooms in each school and ran for 17 h, starting at 18:00 until 11:00 h the next morning.

**Results:**

*Anopheles funestus* was the predominant species collected, forming 93.2% (N = 727) of the entire collection, with peak landing between 06:00 and 07:00 h and continuing until 11:00 h. More than half of the collected *An. funestus* were either fed or gravid, potentially indicative of multiple bloodmeals within each gonotrophic cycle, and had a sporozoite rate of 2.05%.

**Conclusion:**

School children spend up to 10 h of their daytime in schools, reporting between 06:00 and 07:00 h and staying in school until as late as 17:00 h, meaning that they receive potentially infectious mosquito bites during the morning hours in these settings. There is a need to consider vector control approaches targeting schools and other peridomestic spaces in the morning hours when *An. funestus* is active.

## Background

Malaria disproportionately affects sub-Saharan Africa, with > 93% of all malaria cases and deaths occurring in this region. While the disease affects people of all ages, children under five are particularly vulnerable, accounting for over 60% of all malaria deaths worldwide [1]. Malaria case burden extends to the 5–15-year-old age group [1, 2]. Furthermore, malaria infection prevalence is often highest in this age group [3, 4]. Malaria can cause severe anaemia, leading to fatigue and reduced concentration, impacting a child’s learning ability [5–7]. Malaria-related absences from school contribute to lower school attendance and poorer academic performance, further perpetuating the cycle of poverty [8]. Asymptomatic infections, in children aged 5–15 years often go untreated and likely serve as an important reservoir of infection for the entire community [3, 9–11].

There are limited interventions that target control of malaria in school-aged children despite their high burden of malaria infection. Most interventions target the vulnerable groups: under-fives and pregnant women. As children become older and more independent, parents have less control over when they go to bed, where they sleep, and whether they use LLINs [8]. This results in less LLIN coverage and less malaria protection in this age group [12]. School-aged children have greater anti-malarial immunity than younger children, which is acquired through repeated exposure to malaria parasites, gained with repeated infections. Because older children have greater anti-disease immunity, they are often more likely to have asymptomatic parasitaemia than younger children (and adults) and this age group often has the highest parasite prevalence [8, 13, 14]. Schools have been described as a potential avenue for malaria control in children through programmes, such as intermittent preventive treatment in school children (IPT-SC) [15, 16] and seasonal malaria chemoprevention (SMC) [17, 18], due to the logistic feasibility of reaching many eligible children in one place.

On the other hand, schools are potential risk sites for malaria transmission, especially in rural areas with a high disease burden [19]. With the change in mosquito behaviour, they have shifted to resting and biting outside the treated homes to evade the available interventions [20]. Children spend up to 10 h of their daytime at school, including dawn and dusk, when mosquitoes are active, and if schools are not adequately protected against malaria transmission, they can become hotspots for the disease [21].

Ongoing vector surveillance in Siaya County as part of an evaluation of attractive targeted sugar baits (ATSBs) has observed a peak in biting by *Anopheles funestus* at 06:00 h with continued biting into the later hours of the morning (Ochomo et al., unpublished).

Observations have shown that many children in this area consistently arrive at school between 06:00 and 07:00 h. Consequently, it is possible that they are exposed to potentially infectious mosquito bites while seated in class during their prep time and morning lessons. Mosquito abundance and biting behaviour was characterized in primary schools at night and in the morning hours when children would normally be in school.

## Methods

### Study site

The study was conducted in four non-boarding primary schools within Alego-Usonga Sub-County, Siaya County, western Kenya: Bukhoba, Gangu, Kanyaboli, and Gendro. Siaya County is characterized by high, year-round malaria transmission with peak seasons after the long rains (March-June) and short rains (October–November). The primary malaria vectors in the region are *Anopheles gambiae* sensu lato (s.l.) and *An. funestus* s.l. [22, 23], with *An. funestus* being the predominant vector. Siaya County has the second highest malaria prevalence nationally [24], and receives long-lasting insecticidal nets (LLINs) through mass campaigns and routine distribution to pregnant mothers and children through antenatal and child welfare clinics.

**Fig. 1:**
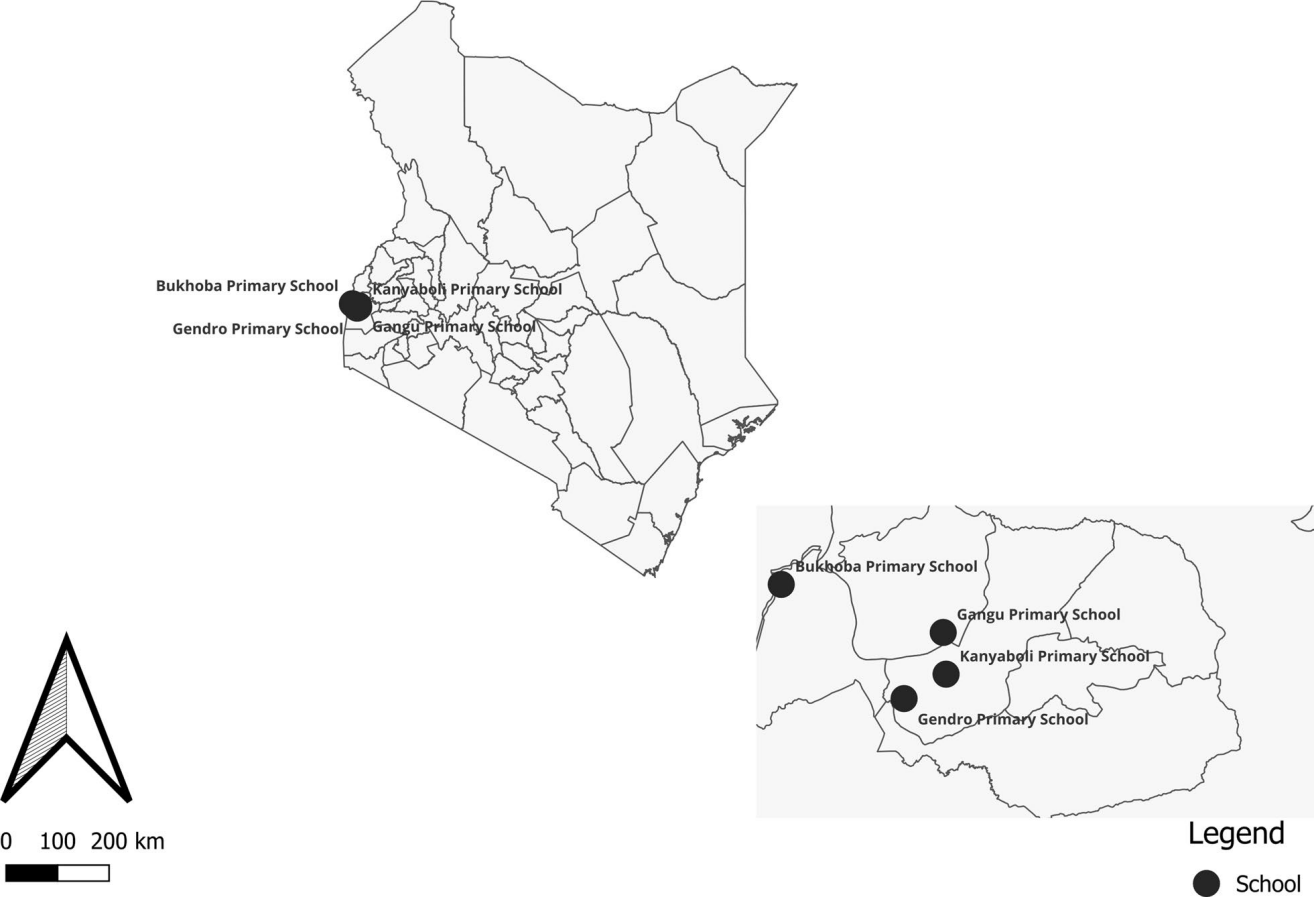
Map of Alego Usonga, Sub County showing the location of the primary schools in this study

Students report to these schools between 06:00 and 07:00 h and leave between 16:00 and 17:00 h. The schools were selected due to the high densities of *Anopheles* mosquitoes observed in the surrounding villages during quarterly mosquito surveillance conducted as part of the ATSB trial [25]. Bukhoba Primary School is situated on a rice plantation, while Gangu, Kanyaboli, and Gendro are located around Lake Kanyaboli, an oxbow lake with surrounding papyrus swamps that provide stable mosquito habitats, particularly for *An. funestus* (Fig. 1).

### Mosquito collection

Mosquitoes were collected using human landing catches (HLC) in five classrooms per school for 2 days in August 2023. Thirty adult males who were members of the community were enrolled and consented to participate as paid mosquito collectors for this study. Prior to the initial collection, the collectors were tested for malaria using the First Response® Malaria Ag. pLDH/HRP2 Combo Card Test rapid diagnostic tests (RDTs), and those who were positive for malaria were treated using artemether/lumefantrine (AL) (Coartem®). The collectors were given daily malaria chemoprophylaxis with doxycycline capsules (100 mg). Mosquito sampling was done hourly from 18:00 h overnight until 11:00 h, indoors and outdoors to match ongoing collections in neighbouring households. Collections were done for the first 45 min and the collectors took a 15-min break within every collection hour to fill out tablet questionnaires indicating the collection date, hour, and location and prepare for the next hour of collection.

During collection, the mosquito collectors wore shorts or rolled-up trousers and clothing that covered their bodies and upper limbs to protect them from unnecessary mosquito bites. Additionally, strict hourly supervision of the collectors was instituted to ensure they did not fall asleep so that they would not get bitten. It is worth noting that, no other mosquito borne infections have been detected in the region [26].

Additionally, HLC is actually a beneficial method of collection for the participants as research conducted in the same region earlier showed lower malaria incidence in HLC volunteers compared to other adults of the same demographic living in the same area [27]. This is because they are usually on malaria prophylaxis during this time. The collectors sat on a chair and collected mosquitoes that landed on their exposed legs using a mouth aspirator. Mosquitoes were aspirated into paper cups labelled using a tablet-generated code indicating the collection date, hour, and location and provided with cotton soaked in a 10% sugar solution. The next morning, mosquitoes were transported to a field laboratory for processing.

Morphological identifications were performed on the *Anopheles* mosquitoes following taxonomic keys [28] to differentiate between *An. funestus* s.l. and *An. gambiae* s.l. and other secondary malaria vectors. They were then separated by species, sex, and abdominal status (blood-fed, non-blood-fed, half-gravid, or gravid) for females and counted by location and hour of collection. A subset of the non-blood fed, non-desiccated *An. gambiae* s.l. and *An. funestus* s.l. females were dissected for parity determination as an indicator of their age and a sample of these were identified to species using PCR [29] and sporozoites detected using enzyme linked immunosorbent assay [30]. Culicine mosquitoes were counted and sexed by location and hour of collection and then discarded.

### Data collection and analysis

The data were collected using CommCare v. 2.52.1. Vector abundance was assessed using descriptive statistics (totals, proportions and means). Line plot visualizations were used to show the trend of vector biting rates over the collection period. All data analysis and visualization tasks were performed in the R statistical computing environment.

## Results

*Anopheles funestus* was the predominant species collected overall (n = 727; 93.2%), and in each of the primary schools (range 78.8% to 95.4, Table 1). Other species collected included *An. gambiae* (n = 49; 6.3%), *An. coustani* (n = 2, 0.25%) and *An. ziemanni* (n = 2, 0.25%). *Anopheles gambiae* s.l. represented 4.7% to 21.2% of collections in the schools, while *An. coustani* and *An. ziemanni* were collected only in Bukhoba primary school. The distance between these schools ranged between 3.14 and 14.16 km. The *An. funestus* eligible for dissection had an overall parity rate of 89.2% (n = 157). The total number of culicines collected comprised of 2247 females and 4 males. The breakdown of the bionomics is presented in Table 1.

**Table 1:** The number (%) of *Anopheles* collected detailing the school categorized by location, species, abdominal, and parity status

School	Gendro	Kanyaboli	Gangu	Bukhoba
Collection location/number collected	66	109	138	467
Indoor	52 (79%)	93 (85%)	96 (70%)	353 (76%)
Outdoor	14 (21%)	16 (15%)	42 (30%)	114 (24%)
Species				
*An. funestus*	52 (79%)	104 (95%)	130 (94%)	441 (94%)
*An. gambiae*	14 (21%)	5 (4.6%)	8 (5.8%)	22 (4.7%)
*An. coustani*	0	0	0	2 (0.4%)
*An. ziemanni*	0	0	0	2 (0.4%)
Abdominal status				
Fed	28 (42%)	69 (63%)	49 (36%)	224 (48%)
Gravid	6 (9.1%)	11 (10%)	13 (9.4%)	15 (3.2%)
Half gravid	1 (1.5%)		2 (1.4%)	6 (1.3%)
Unfed	31 (47%)	29 (27%)	74 (54%)	222 (48%)
Parity (*Anopheles funestus* only)/number dissected	17	10	33	97
Nulliparous	2 (11.8%)	1 (10%)	0	14 (14.4%)
Parous	15 (88.2%)	9 (90%)	33 (100%)	83 (85.6%)
Culicines collected	Females			
Indoor	357	29	108	537
Outdoor	480	50	75	606
	Males			
Indoor	0	0	0	0
Outdoor	1	0	1	2

All of the *An. funestus* s.l. samples were identified as *An. funestus* sensu stricto (s.s.) (N = 727) by PCR and had a sporozoite rate of 2.05% while 98% (n = 49) of *An. gambiae* s.l. were identified as *An. arabiensis* and the rest as *An. gambiae* s.s. None of the *An. gambiae* s.l. tested positive for sporozoite infectivity.

**Fig. 2:**
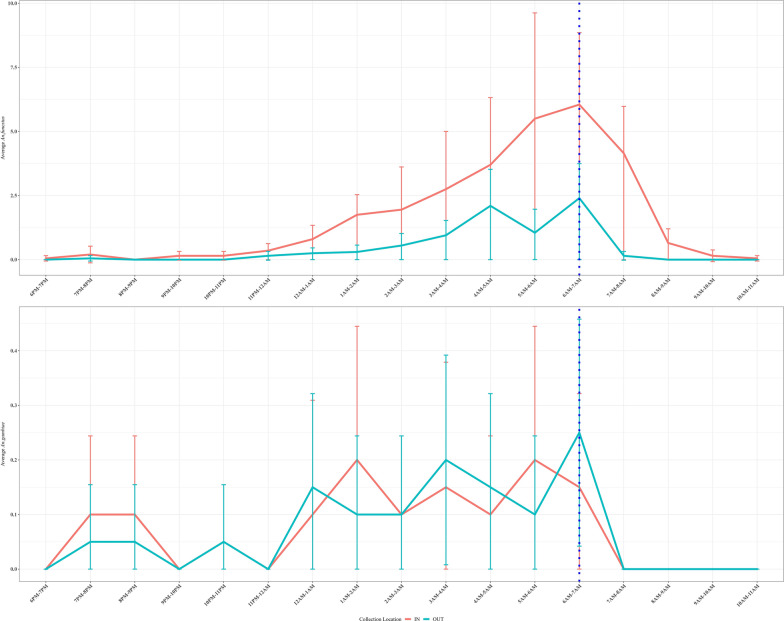
The mean number of *An. funestus* and *An. gambiae* s.l. collected hourly between 18:00 to 11:00 h

*Anopheles funestus* landing rates indoors increased steadily between 23:00 and midnight, peaking between 06:00 and 07:00 h and declined rapidly, with biting continuing at lower levels until the end of collections at 11:00 h. Inside the classrooms, collectors received an average of 1.8 *An. funestus* landings between 18:00 and 07:00 h and 1.25 bites between 07:00 and 11:00 h. Outdoors, they received an average of 0.6 landings between 18:00 and 07:00 h and only an average of 0.04 landings after 07:00 h. The collectors received an average of 0.1 *An. arabiensis* landings indoors between 18:00 and 07:00 h and 0.09 landings outdoors. *Anopheles arabiensis* did not land on the collectors after 07:00 h. In outdoor settings, *An. funestus* exhibited two distinct peaks, the first between 04:00 and 05:00 h and the second and a slightly larger peak coincident with the indoor peak between 06:00 and 07:00 h, but no landing was recorded after 09:00 h. *Anopheles gambiae* densities were much lower (averaging 0.2 mosquitoes per hour at peak) with no discernable differences in density between the indoor and outdoor collections. *Anopheles gambiae* activity was high between 23:00 and 08:00 h with no clear peaks indoors or outdoors (Fig. 2). Although a few *An. gambiae* were collected between 08:00 and 09:00 h, landing by this species largely ceased after 07:00 h.

## Discussion

This study describes the collection of high densities of *Anopheles* mosquitoes, 93.2% of which were *An. funestus*, in primary schools in a malaria endemic area of western Kenya. Despite being only a 2-day sampling effort in August 2023, which is not peak mosquito season, the study yielded high densities of *An. funestus* averaging > 17 *An. funestus* females per collector, per hour. *Anopheles funestus* is one of the primary vectors in the area, having re-emerged about a decade ago [23], but it has a heterogeneous distribution evidenced by the collection of more than half of the mosquitoes in one school. Surprisingly, more than half of all the mosquitoes collected landing on the collectors were either already fed or gravid, suggesting that the vector has a repeat feeding behaviour, which has been documented in *An. arabiensis* in Zambia [31] but not in *An. funestus*, and certainly not at this scale. Lower densities of *An. gambiae* were observed, while only two each of *An. coustani* and *An. ziemanni* were collected. Importantly, *An. funestus* mosquitoes were collected indoors and outdoors, landing on HLC volunteers in high numbers up to 9 a.m. After 9 a.m., no mosquitoes were collected outdoors, with indoor mosquito numbers tapering off until 11 a.m., indicating that school children are at risk of malaria infection during the day.

School-based studies of malaria have been aimed at schools as avenues for conducting surveillance of malaria prevalence and incidence [14, 32–35] or schools as delivery mechanisms for chemoprevention through seasonal malaria chemoprevention [17] and intermittent preventive therapy in school children (iPTSC) [15, 36, 37]. This is informed by the often-higher parasite prevalence in children between the ages of 5–15, considered the primary school-going age, followed by children < 5 years of age, even though children < 5 years of age have the greatest burden in terms of morbidity and mortality due to an underdeveloped immune system [1]. However, this is the first investigation of *Anopheles* vectors as a contributor to malaria transmission within these school settings. Importantly, because children in high transmission settings have already developed immunity by this age, they remain as reservoirs of malaria infection in their communities because of greater anti-disease immunity to malaria than the younger children, but lower anti-parasite immunity than adults, thus school-aged children are at higher risk of asymptomatic parasitaemia [10, 11]. Because of this, school-aged children are less likely to seek treatment than younger children and therefore less likely to have the parasitaemia cleared with antimalaria drugs, and are also likely to have the poorest usage of nets [8, 38, 39] thus remaining important reservoirs of infection in the communities.

Several studies have reported changes in *Anopheles* vector biting behaviour, with changing preference towards early evening and late morning biting when people are often not under the protection of their bed nets [40–43]. Also, insecticide resistance in these mosquito populations could be leading to the survival of these mosquitoes despite exposure to pyrethroid insecticides [44]. Additionally, the early morning biting observed here points to a change in behaviour to avoid bednets. Entomological surveillance studies currently being conducted as part of a randomized control trial to evaluate the epidemiological efficacy of attractive targeted sugar baits in this study area (ClinicalTrials.gov ID NCT05219565) have reported late morning biting in *An. funestus* (Omondi et al., unpublished). These studies are coupled with observations of human activities and behaviour to understand the opportunities for human vector contact leading to malaria transmission. School children spend a significant portion of their time in schools, reporting between 06:00 and 07:00 h and staying in school until as late as 17:00 h.

Interestingly, the peak in biting was at 06:00 h, just as the children would normally be arriving at school. This means that children sitting in these structures are potentially being bitten during their morning classes as they sit still and pay attention during their lessons. Additionally, the high parity rates observed in all four schools suggests that a large proportion of these vectors had had prior bloodmeals and were potentially infectious. Indeed, 2.05% of the tested *An. funestus* were found to have *Plasmodium falciparum* sporozoites detected.

This study highlights the need to consider vector control approaches in schools and other peridomestic spaces where people are likely to be spending time when *An. funestus* mosquitoes are active during the day. The use of chemotherapeutic/chemoprophylactic approaches in combination with vector control in schools has the potential to reach a large asymptomatic reservoir of infectious individuals at a low logistical cost in line with the recent WHO’s recommendation for IPTsc in areas of moderate to high perennial or seasonal malaria transmission [43]. In particular, significant cost-benefit may be achieved with vector control approaches such as indoor residual spraying or spatial repellents protecting a large number of children attending school.

The study has several limitations, including a short and focused data collection period in August 2023, which may not capture the full seasonal variations in mosquito behaviours and malaria transmission. Using human landing catches (HLC) exposes collectors to potential health risks and may not accurately represent outdoor or animal-host-seeking mosquito behaviours. While treated and provided with chemoprophylaxis, the reliance on community members as collectors raises ethical concerns about their potential exposure to infectious mosquito bites. The study does not include a control group, making attributing changes in vector abundance challenging. Finally, the generalizability of the findings to other regions with different ecological characteristics is uncertain, given the specific conditions in Siaya County, Kenya.

## Conclusion

This study was conducted in a highly endemic malaria setting in Siaya, where there is an abundance of *An. funestus.* These vectors have been observed to shift their biting patters, peaking between 06:00 and 07:00 h, coincident with when children generally report to non-boarding primary schools. *An. funestus* were observed to bite as late as 11:00 h meaning that children are receiving potentially infectious mosquito bites during the morning hours in these settings. For this reason, it is important to consider vector control approaches targeting schools and other peridomestic spaces in the morning hours when *An. funestus* is active.

## Data Availability

All the study data is available and can be shared upon request to the corresponding author.
